# Thermal Processing Effects on Antioxidant Properties, Physicochemical, and Sensory Characteristics of Whey Açaí (*Euterpe oleracea* Mart.) Beverages

**DOI:** 10.3390/foods14244319

**Published:** 2025-12-15

**Authors:** Vitor Luiz M. Silva, Ana Rafaela S. Leal, Maria Clara S. Andrade, Roberta O. R. Ribeiro, Fabricio O. Silva, Eliane T. Mársico, Igor A. Rodrigues, Carlos A. Conte-Junior, Carla S. Carneiro

**Affiliations:** 1 Graduate Program in Veterinary Hygiene (PPGHV), Faculty of Veterinary Medicine, Federal Fluminense University (UFF), Niterói 24230-340, RJ, Brazil; 2 Faculty of Veterinary Medicine, Cesmac University Center, Maceió 57051-160, AL, Brazil; 3 Faculty of Pharmacy, Federal University of Rio de Janeiro (UFRJ), Rio de Janeiro 21941-902, RJ, Brazil; 4 Municipal Institute for Health Surveillance, Zoonosis Control, and Agricultural Inspection—IVISA-RIO, Rio de Janeiro 20230-070, RJ, Brazil; 5 Department of Food Technology, Federal Fluminense University (UFF), Niterói 24230-340, RJ, Brazil; 6 Graduate Program in Pharmaceutical Sciences, School of Pharmacy, Federal University of Rio de Janeiro (UFRJ), Rio de Janeiro 21941-902, RJ, Brazil; 7 Department of Biochemistry, Chemistry Institute, Federal University of Rio de Janeiro (UFRJ), Rio de Janeiro 21941-909, RJ, Brazil

**Keywords:** Açaí, whey, thermal processing, total phenolic, anthocyanins, antioxidant capacity

## Abstract

The development of a mixed beverage combining açaí pulp and whey unites the antioxidant potential of açaí with the nutritional and functional properties of whey, offering a promising option for health-oriented food products. This study evaluated the effects of thermal processing on the antioxidant properties, physicochemical parameters, and sensory acceptance of an açaí (*Euterpe oleracea* Mart.) pulp–whey-based beverage (APWBB). Samples were heated at 75 °C, 80 °C, and 90 °C for 15, 60, and 300 s, resulting in nine treatments (T1–T9). Thermal processing had little influence on the beverage’s physicochemical parameters; however, total phenolic content and total anthocyanins progressively decreased with increasing temperature and heating time. Losses reached 44.30 to 16.30 mg GAE/100 mL (phenolics) and 10.03 to 6.30 mg/100 mL (anthocyanins) between the control and the most intense treatment, showing a linear reduction pattern. These decreases were strongly correlated with reductions in antioxidant capacity (FRAP, DPPH, and ABTS), demonstrating the sensitivity of bioactive compounds to heat. Sensory evaluation revealed no significant differences among treatments, and the beverage showed high acceptance (71–80%) and positive purchase intention, highlighting its stability and market potential. Overall, the combination of açaí and whey represents a promising matrix for developing functional beverages, provided that suitable thermal parameters are adopted to minimize bioactive compound degradation.

## 1. Introduction

The açaí (*Euterpe oleracea* Mart.) berry is a fruit native to the Amazon region situated in Brazil, which has gained international importance due to its hypocholesterolemic, anti-tumor, anti-inflammatory, anti-genotoxic, and antioxidant activity, associated with its phytochemical composition, mainly phenolics compounds such as anthocyanins and phenolics acids [1], that present effect on the prevention or progression of metabolic diseases, such as diabetes, renal ischemia and nonalcoholic fatty liver disease [2,3,4,5]. Thus, this fruit has become a target of investigations focused on analyzing and assessing its capacities [6,7]. Moreover, açaí berries and their products are attractive due to their potential to deliver sensory attributes that appeal to consumers [8,9,10]. This fruit has been increasingly used in new food formulations [11,12,13,14,15,16]. The mix of açaí and whey is an emerging trend in the market, aiming to merge the antioxidant properties of açai with the nutritional and functional benefits of whey.

In this sense, although cheese whey had been considered the main waste by-product in the dairy industry, interest in the use of whey has increased owing to its high nutritional and functional properties [17]. Its composition consists of lactose and proteins with elevated biological value [18,19], which, after ultrafiltration processing, positions this product as a remarkable product in the global market [20]. The Whey permeate can serve as an industrial-scale source of naturally occurring functional peptides and oligosaccharides [21,22], which possess antioxidant, antihypertensive, and antimicrobial activities [23].

Therefore, the nutritional and functional characteristics of açaí and whey make them interesting ingredients for the formulation and development of novel food products [17,24]. Acai pulp can be incorporated to enrich dairy products and other food matrices. At the same time, whey serves as a functional ingredient that can improve protein value and contribute to technological properties such as texture, viscosity, and gelation [25,26]. Beyond their technological applications, the widespread availability of information on the health benefits of these ingredients contributes to consumers perceiving functional foods as tools for enhancing health and well-being [16,27,28,29].

However, these products must undergo heat treatments for production on a plant scale. Furthermore, it is known that phytochemicals, such as anthocyanins and phenolics, are susceptible to degradation during processing [30,31]. Temperature and time are considered the key factors influencing the degradation rate of such molecules [32]. During thermal processing, high temperatures may have undesirable effects on these products, affecting their sensory traits and, consequently, their acceptance [33]. Despite conventional thermal processing still being the most commonly used preservation technique in dairy products with fruits, there is a lack of data on its influence on sensory traits and on phenolic compound degradation during thermal treatment.

Considering that conventional thermal processing remains the most widely used preservation method for dairy products and fruit-based beverages, it is essential to obtain data on its effects on sensory properties, as well as its potential to degrade phenolic compounds and reduce antioxidant capacity. This need is even more relevant for emerging formulations that combine fruits rich in phenolic compounds with dairy matrices.

This study aimed to evaluate the influence of thermal processing effect on antioxidant properties, physicochemical parameters, and sensory characteristics in açaí (*Euterpe oleracea* Mart.) pulp-whey-based beverage.

## 2. Materials and Methods

### 2.1. Açaí Pulp-Whey-Based Beverage (APWBB) Manufacture

The *Euterpe oleracea* Mart. (Açaí) pulp purchased from the retail market (Rio de Janeiro, RJ, Brazil) was extracted with the addition of water at a ratio of 1:1. The content of total solids of the pulp obtained ranged from 8 to 11%. To produce the APWBB, açaí pulp and whey were mixed in a 1:1 ratio under constant agitation, and 10% sucrose was then added to the formulation.

### 2.2. Thermal Treatment

APWBB were packaged in glass bottles, sterilized (250 mL), and submitted to 9 different heat Treatments (T1-T9). The samples were submitted to the following heat treatments: 75 °C for 15 s (T1); 75 °C for 60 s (T2); 75 °C for 300 s (T3); 80 °C for 15 s (T4); 80 °C for 60 s (T5); 80 °C for 300 s (T6); 90 °C 15 s (T7); 90 °C 60 s (T8) and, 90 °C 300 s (T9). These binomial treatments were based on the pasteurization process used in juice industries and pulps to eliminate microorganisms and to inactivate enzymes [31]. The treatments were performed with the glass flasks fully immersed in a water bath/heat exchanger with thermostatic control. Immediately after the immersion period, all flasks were transferred to an ice-water bath to rapidly reduce the internal temperature to refrigeration levels (8 °C), at which they were maintained until analysis. In addition, a sample of açaí pulp not heat-treated was analyzed and used as an untreated control.

### 2.3. Titratable Acidity, pH, and Total Soluble Solids

The Titratable Acidity (TA) was performed using method No. 945.26 of Official Methods of Analysis of AOAC International [34], with 0.1 M NaOH to determine the equivalence point by pH measurement between 8.2 and 8.4, and expressed as citric acid. The pH was determined according to method No. 981.12 [34]. Total Soluble Solids (TSS) were measured using an optical refractometer (Model ABBE; Biobrix^®^, Delhi, India), and results were expressed as degrees Brix, according to method No. 932.14 [34].

### 2.4. Determination of Total Phenolic Content

The Folin–Ciocalteu method, as described by Singleton et al. [35], was modified. Sample (1 mL) was added to a volumetric flask, and the flask was filled to 100 mL with distilled water. After 10 min in an ultrasound bath, the samples were stored at 4 °C overnight (12 h) and then returned to the ultrasound bath for 10 min. Then, filtered through a membrane with a 0.45 µm pore size. 500 µL were used to perform the reaction in 2.5 mL of Folin-Ciocalteu during 1 min at 2400 rpm on a vortex (Certomat^®^, Goettingen, Germany). Thereafter, 2 mL of Na_2_CO_3_ solution (7.5%) was added, and the mixture was homogenized. All the samples were kept in the dark for 2 h before absorbance was measured at 760 nm in a UV-1800 Spectrophotometer (Shimadzu, Kyoto, Japan). For the blank sample, 0.5 mL of distilled water was used in place of the extract. The calibration curve was plotted over a range of 9.31 to 49.36 mg of Gallic Acid Equivalents (GAE) per 100 mL of mixed beverage, and the results were determined from the calibration curve’s regression equation (y = 19.758x − 0.0091; R^2^ = 0.9861). The results are expressed as mg GAE/100 mL.

### 2.5. Total Anthocyanin Content

Anthocyanin content was estimated by the method described by Giusti and Wrolstad [36]. The absorbance difference at pH 1.0 and pH 4.5 was directly proportional to anthocyanin concentration. The calculation was based on cyanidin-3-glucoside. Two aliquots from each sample were used: one was homogenized with 25 mM KCl, pH 1.0, and the other with 0.4 M sodium acetate, pH 4.5. After 60 min at 25 °C, the samples were centrifuged at 3000× *g* for 20 min at 4 °C (Sorvall ST 16R, Thermo Scientific, Waltham, MA, USA). Absorbance (A) was measured at 520 and 700 nm using a UV-1800 spectrophotometer (Shimadzu^®^, Kyoto, Japan). The dilution factor was pre-determined when the absorbance value at 520 nm of the homogenate at pH 1.0 was between 0.2 and 1.4. The blank solution was distilled water, and the results were expressed as cyanidin-3-glucoside equivalents, considering a molar absorptivity coefficient of 26,900 L.mol^−1^.cm^−1^ and the results were calculated according to Equation (1):

Total anthocyanin content

(Cyanidin-3-glucoside Equivalents, mg/L) = (A × MW × DF × 1000)/(ε × 1)(1)

where A = (*A*_520nm_ − *A*_700nm_) pH 1.0 − (*A*_520nm_ − *A*_700nm_) pH 4.5; MW (molecular weight) = 449.2 g/mol for cyanidin-3-glucoside (cyd-3-glu); DF = dilution factor; l = light path length in cm; ε = 26,900 molar extinction coefficient, in L mol^−1^ cm^−1^, for cyd-3-glu; and 1000 = conversion from g to mg.

### 2.6. Determination of Antioxidant Activity by FRAP, DPPH, and ABTS

Extraction procedures for the analysis of antioxidant capacity were carried out according to the method described by Muller et al. [37] using a UV-1800 Spectrophotometer (Shimadzu, Kyoto, Japan). Blank, standards, and extracts were also measured.

The antioxidant capacity was determined according the method Benzie and Strain [38], with minor modifications by Cesar et al. [39] which measures its ability to reduce Fe(III)-2,4,6-Tri(2-pyridyl)-s-triazine (TPTZ) complex to Fe(II)-TPTZ, the resulting intense blue color is related to the amount of reductant (antioxidant) present. Absorbance was measured at 593 nm 4 min after 1 mL of a ten-fold dilution of the samples was added to 3 mL of Fe (III)-TPTZ, and ferric reducing antioxidant potential (FRAP value) was quantified from a standard curve previously constructed using a stock solution. The results were expressed as micromoles of ferrous equivalents per gram fresh weight.

DPPH values were determined by the method of Brand-Williams et al. [40]. In this method, considered highly applicable, the 2,2-diphenyl-1-picrylhydrazyl radical (DPPH•) reacts with an antioxidant that can donate hydrogen and reduce DPPH. Modifications were implemented as described by Kim et al. [41]. The decrease in the absorbance of 100 µM DPPH• radical (2.9 mL) dissolved in 80% methanol was evaluated at 515 nm 30 min after the addition of each extract. The results are expressed as µMol/g of TEAC (Trolox-equivalent antioxidant capacity).

The antioxidant activity using the ABTS method (2,2′-azino-bis(3-ethylbenzthiazoline-6-sulphonic acid) was determined as described by Re et al. [42]. The process is based on the deactivation of the antioxidant radical cation ABTS•+, which is measured by the decrease in absorbance at 734 nm. Aliquots of the sample solution or extract were diluted with ABTS solution, and absorbance was monitored 7 min after the addition of the extract (UV-1800 Spectrophotometer, Shimadzu, Kyoto, Japan). The results are expressed as µMol/g of TEAC.

### 2.7. Sensory Analysis

A total of 102 valid participants took part in the study. Before participating in the sensory evaluation of the APWBB, all volunteers confirmed that they had no allergies or intolerances to any of the beverage’s ingredients. The study was conducted in accordance with the Declaration of Helsinki, and the protocol was approved by the Ethics Committee of the Universidade Federal do Rio de Janeiro (approval code: 40810215.1.0000.5257) in 2015. Each participant was provided with approximately 30 mL of the APWBB at 4 °C. For this analysis, an incomplete block experimental design was used, and the samples were coded with random three-digit numbers. A discriminative method (Duo-Trio Test) was used to assess whether there was a difference overall (flavor, color, aroma, appearance, viscosity, texture, and other attributes relevant to the taster) between the samples (T1–T9). In this test, it was necessary for the evaluators to choose which sample was most similar to the reference (control) sample. In addition, two affective sensory teste (also with 102 panelists) was performed: 9-points hedonic scale (1 = “dislike extremely”, 2 = “dislike very much”, 3 = “dislike moderately”, 4 = “dislike slightly”, 5 = “neither like nor dislike”, 6 = “like slightly”, 7 = “like moderately”, 8 = “like very much” and 9 = “like extremely”) and, 9-points attitude scale (1 = “I only drink if forced”, 2 = “I only drink if I could not choose a better food”, 3 = “I rarely drink”, 4 = “I did not like, but drink occasionally”, 5 = “I drink if available, but I would not strive me to obtain it”, 6 = “I liked and drink occasionally”, 7 = “I drink often”, 8 = “drink very often” and 9 = “I drink whenever I have a chance”), to estimate acceptance and frequency of consumption, respectively.

### 2.8. Statistical Analysis

Data were expressed as the means ± S.D. (standard deviation). Differences between treatments were evaluated using a one-way ANOVA at the 95% confidence level (*p* < 0.05). Post hoc pairwise comparisons of means were performed using Tukey’s test. Pearson correlation analysis of the parameters total phenolic content, total anthocyanin content, and antioxidant activity by FRAP, DPPH, and ABTS was performed, and correlation coefficients (r) were calculated. All analyses were performed using XLSTAT software (version 2015; Addinsoft, Paris, France). All the analyses were performed in triplicate.

## 3. Results and Discussion

### 3.1. Physicochemical Parameters

The physicochemical characteristics of the açaí pulp and açaí pulp-whey-based beverage (APWBB) submitted to different thermal treatments are shown in Table 1.

Regarding pH, total acidity (TA), and total soluble solids (TSS), relatively small differences were observed among the various treatments. No differences in total acidity were observed between treatments. This indicates that the concentrations of organic acids in the beverage remained unaltered as a constant net balance. Similar effects were observed by Cesar et al. [39] for clarified açaí juice.

The pH value of açaí pulp is consistent with those reported by others. Eto et al. [43] reported a pH of 4.79 for açaí pulp; Carvalho et al. [6] presented pH values ranging from 5.03 to 5.23 for açaí genotypes and commercial pulps (lyophilized samples); and Aires et al. [44] reported a pH range of 5.10 to 5.35. The pH of APWBB was higher than that of the fruit pulp due to mixing with whey. The pH of the whey used to prepare APWBB in this experiment was 6.47 ± 0.02.

The pH values of the APWBB ranged from 5.00 to 5.14, showing relatively small differences between treatments. The lowest average values were observed in treatments T2 and T3 (pH 5.00), while the highest was observed in T8 (pH 5.14), although all treatments remained within a narrow range. The pH and titratable acidity remained stable across different temperatures and processing times. Overall, the heat treatment did not degrade the organic acids present, and these parameters were not affected. This low variability indicates that the açaí-whey system has a high buffering capacity, a behavior expected in dairy beverages due to the presence of proteins and minerals that resist changes in pH and total acidity during heating [45].

The total acidity results obtained here for the acai pulp agree with Brazilian legal standards, which determine 0.27% as the maximum value for this raw material. The total acidity did not vary between the control and treatments, remaining at 0.1%. Similarly to our work, Salvia-Trujillo et al. [46] found no significant differences in total acidity of heat-treated fruit juice–milk beverages. These authors blended orange, kiwi, mango, and pineapple with whole or skim milk, then thermally treated the mixture at 90 °C for 1 min. They observed acidity in fruit juice–skim milk and fruit juice–whole milk beverages corresponding to 1.1% and 1.33% of the citric acid concentration, respectively.

Regarding the TSS of the acai pulp, Sousa et al. [47] detected a value of 3.20 °Brix in a study of açaí pulp in natura, Neves et al. [48] detected 4.0 °Brix in açaí pulp, and Santos et al. [48] detected 3.40 °Brix, similar to the values obtained in the present study. In the present study, the increase in TSS in APWBB compared to açaí pulp was related to the addition of whey (lactose and peptides) to the formulation. A slight reduction in total soluble solids (TSS) was observed between treatments T2 and T7, attributed to slight reductions in solids resulting from chemical reactions induced by heating, such as the degradation of sugars or the initiation of Maillard reactions. This variation, however, is minimal and does not represent a significant change in the beverage composition. In general, mild heat treatment does not significantly affect the soluble solids content of fruit-based beverages, with variations remaining within the 0.3–1.0 °Brix range [49,50]. The results of the physical-chemical analyses indicate good stability of the beverages to heat treatment.

### 3.2. Temperature and Time Effect on the Total Phenolic Content and Total Anthocyanin

The total phenolic content, total anthocyanins, and antioxidant capacity obtained in the present study are shown in Table 2.

The contents of phenolic compounds and anthocyanins in the açaí pulp were 75.51 ± 2.14 mg GAE/100 mL and 14.95 ± 0.14 mg/100 mL, respectively. Kuskoski et al. [51] detected a total phenolic content of 136.80 mg/100 mL and anthocyanin values of 22.80 ± 0.8 mg/100 mL in açaí pulp. Other authors [48,52] have reported total phenolic values ranging from 182.95 to 598.55 mg/100 mL and total anthocyanin values ranging from 13.93 to 54.18 mg/100 mL for commercial açaí pulp. The lower values found in the study were due to dilution of the pulp (1:1) to prepare the beverage. In addition, it is relevant to note that compounds vary among açaí fruit cultivars, species, and commercial brands of açaí pulp [53,54,55].

The beverage developed in the present study was pasteurized at different processing times and temperatures. Pasteurization is the exposure of the pulp or juice to temperatures below 100 °C for a few seconds. This technique inactivates enzymes and eliminates heat-sensitive microorganisms, thereby extending the product’s shelf life [56,57]. However, the degradation of nutrients and functional compounds at high temperatures remains a key issue influencing the quality and functionality of the final product [58,59,60]. Heating leads to oxidation and hydrolysis of phenolics and anthocyanins, reducing their stability. In this whey-açaí matrix, heat also promotes polymerization and interactions with proteins and sugars, decreasing the measurable content of these compounds [30]. The present results showed that the total phenolic and anthocyanin content decreased significantly (*p* < 0.05) with increasing temperature in the açaí pulp-whey-based beverage.

The analysis of total phenolics in APWBB resulted in contents of 44.30 ± 2.92 to 16.30 ± 0.00 mg GAE/100 mL between control and T9 (90 °C for 300 s). The concentration of total anthocyanin of control and T9 ranged from 14.95 ± 0.14 to 6.30 ± 0.07 mg/100 mL, respectively. The thermal processing of APWBB resulted in a 60.82% loss of total phenolic content (25.30 mg GAE/100 mL) and 33.40% of total anthocyanin content (3.16 mg/100 mL) between the T1 sample (75 °C for 15 s) and the T9 sample submitted to a longer processing time and to a higher temperature (90 °C for 300 s).

To date, there are no data available about the influence of thermal treatment on total phenolics and anthocyanins in dairy products with fruit. However, several authors have reported the potential thermal effect on these compounds in fruit beverages. Askin et al. [61] reported a reduction in total anthocyanins in black mulberry (Morus nigra) juice during heat treatment at temperatures similar to those used here (a 39–51% loss). Hooshyar et al. [62] demonstrated that the pasteurization process significantly decreased (*p* < 0.05) the total phenolic content of red grape, pomegranate, and sour cherry juices. Igual et al. [63] observed a 14.64% depletion of total phenolic and flavonoid content in grape juice subjected to standard pasteurization. In a study of holding time and mild heat temperature, Sew et al. [64] showed that under constant mild heat conditions (50 °C for 20 min), total phenolic content in pineapple juice decreased from 84.19 to 75.62%. Likewise, total phenolic content decreased from 72.80% to 57.37% as the holding time increased from 10 to 30 min at 55 °C.

Mikkelsen and Poll [65] studied the decomposition of anthocyanins during the processing of black currant (BC) juice and observed losses of 25% and 30% of anthocyanins. Dobson et al. [66] demonstrated that pasteurization reduced TAC by 15% in BC juice (43.4 to 36.7 mg / 100 mL) and 15% to 18% in mixed juices prepared with addition of apple, persimmon, and peach juices (5.3 to 5.6 to 4.5 to 4.6 mg/100 mL), comparable to the losses previously found for pasteurization during BC juice processing described by Mikkelsen and Poll [65]. In this study, the heat treatment (98 °C, 60 s) was the most destructive stage of processing. Albarici and Pessoa et al. [56] also evaluated the effects of temperature and time on anthocyanin stability in açai drinks at 0 °C, 25 °C, and 40 °C. They noted that, at all tested temperatures, the decrease in anthocyanins over time was linear. When the product was stored at 40 °C, anthocyanin degradation was 1.8 times faster than at 25 °C and 15 times faster than at 0 °C. In the present study, the degradation of total phenolics and anthocyanins in the APWBB was also linear.

### 3.3. Correlation Between Antioxidant Capacity Contents of Total Phenolics and Total Anthocyanins

Pearson’s correlation analysis was used to assess the relationships between the bioactive compounds and antioxidant activity across the different methods. Phenolic compounds (FRAP: r = 0.96; DPPH: r = 0.84, and ABTS: r = 0.91) and total anthocyanins (FRAP: r = 0.98; DPPH: r = 0.92, and ABTS: r = 0.96) were strongly correlated with the antioxidant activity of the APWBB (*p* < 0.05).

Some authors have demonstrated a strong positive correlation among total phenolic content, anthocyanins, and the antioxidant capacity of fruits and fruit juices. Similarly to the present study, Rufino et al. [52] found a positive and significant correlation between polyphenols and antioxidant capacity using the ABTS (r = 0.92) and FRAP (r = 0.89) methods in 18 non-traditional Brazilian tropical fruits, including açaí. Rigon and Noreña [67] reported a high positive correlation between the phenolic compounds and the antioxidant activity of blackberry juice (DPPH: r = 0.86; ABTS: r = 0.95). In a study of a new isotonic beverage with lemon and açaí, Gironés-Vilaplana et al. [68] observed that the concentration of total phenolics was strongly correlated with the two antioxidant capacities assayed (r = 0.980 and 0.830, with *p* < 0.001; for ABTS and DPPH, respectively).

These methods of evaluating antioxidant capacity also showed a high positive correlation (*p* < 0.05) with r = 0.94 for FRAP/DPPH, r = 0.98 for DPPH/ABST, and r = 0.98 for ABTS/FRAP. This means that the analytical methods used produced very similar responses and can be used without distinction to quantify the antioxidant capacity of this kind of beverage.

### 3.4. Results of Sensory Analysis

No sensory difference (*p* > 0.05) was observed among the treatments using the Duo-Trio test. These results suggest that thermal processing did not influence the sensory characteristics of the APWBB. After observing that the heat treatment did not affect the beverage’s sensory attributes, the evaluation of overall acceptance and purchase intention of the product was conducted. This was done because there are no reports on a similar product development market (with this formulation: Açaí Pulp-Whey). The frequency of scores assigned to overall acceptance indicated that 80% of the evaluators demonstrated satisfactory acceptance, with score 6 (like slightly) predominant on the hedonic scale. Moreover, only 14% of the evaluators did not accept the APWBB, with 1–4 on the hedonic scale, and 6% of the evaluators claimed to be indifferent to the APWBB. Consumers were mainly female, with a mean age of 25 years; 87.3% female and 12.7% male; and students with the highest educational degree as a high school diploma or graduate student.

In the present study, the mean overall acceptance score was 6.2. This result was higher than the overall liking between 4.7 and 5.6 for three açaí-based juices reported by Menezes et al. [10]. Compared with the 40% açaí fruit juice evaluated by Sabbe et al. [69], which received significantly higher mean scores for overall liking, the present results are relatively consistent, as the beverage in this study was prepared with 50% açaí pulp. In addition, frozen açaí dessert showed similar overall acceptance scores of 6.8–7.8 [16].

The overall acceptance obtained in the present study aligns well with values reported for other açaí-based products. For instance, the 40% açaí juice evaluated by Sabbe et al. [69] received significantly higher mean acceptance scores, which is consistent with our findings, given that the beverage in this study contained an even higher açaí pulp concentration (50%). Similarly, the açaí frozen dessert assessed by Silva et al. [16] showed comparable acceptance levels, ranging from 6.8 to 7.8. In contrast, Menezes et al. [10] reported lower overall acceptance scores (4.7–5.6) for three different açaí-based juices, highlighting the influence of formulation and product type on consumer perception.

Regarding purchase intention, it was observed that 70% of evaluators indicated they would like to consume the APWBB, with scores between 6 and 9 on the hedonic scale. In comparison, 14% would only consume the APWBB when the product is available; in other words, they do not intend to seek it out on the market to buy it. 16% did not consume, with scores of 1–4 on the hedonic scale. The average purchase intention score in this study was 6.1. The high purchase intention can be attributed to consumers’ positive perceptions of açaí’s and whey’s functional properties, as well as the beverage’s favorable sensory attributes, such as taste and palatability. In contrast, Sabbe et al. [69] reported lower purchase intention scores for açaí-based products, ranging from 2.3 to 4.7. Another study on açaí juice found that only formulations with lower açaí concentrations achieved higher purchase intention [70,71]. According to these authors, health claims and information may slightly increase purchase intention, but sensory quality remains the primary factor.

## 4. Conclusions

This study demonstrated that the pasteurization conditions evaluated had little impact on the physicochemical parameters of the whey-açaí beverage. However, the total phenolic and anthocyanin content of the beverage progressively decreased with increasing temperature and pasteurization time, evidencing a strong sensitivity of these compounds to heat treatment. The observed reduction was linear, reinforcing the cumulative effect of heat on bioactive compounds. Furthermore, phenolics and anthocyanins showed a strong correlation with antioxidant capacity, indicating that their degradation directly impacts the antioxidant potential of the beverage. Regarding sensory characteristics, the whey-açaí beverage showed high acceptance and purchase intention, with no sensory differences between treatments, demonstrating good product stability. With 80% sensory acceptance, it showed potential for market insertion. Taken together, these results demonstrate that the combination of açaí and whey constitutes a promising matrix for the development of functional beverages, provided that appropriate thermal parameters are adopted to minimize the degradation of bioactive compounds.

## Figures and Tables

**Table 1:** Physicochemical characteristics of açaí pulp and açaí pulp–whey-based beverages (APWBB) under different heat-treatment conditions.

	Temperature	Time	pH	TA	TSS
	°C	s		%	°Brix, 25 °C
Acaí pulp	N/A	N/A	4.46 ± 0.01	0.14 ± 0.00	3.22 ± 0.00
Control	Unheated	N/A	5.04 ± 0.01 ^a,c^	0.11 ± 0.01 ^a^	5.08 ± 0.00 ^a^
Treatments					
T1	75	15	5.11 ± 0.03 ^b,e^	0.10 ± 0.01 ^a^	5.01 ± 0.12 ^a^
T2	75	60	5.00 ± 0.02 ^c^	0.10 ± 0.00 ^a^	4.85 ± 0.06 ^a^
T3	75	300	5.00 ± 0.02 ^c^	0.10 ± 0.00 ^a^	4.91 ± 0.06 ^a^
T4	80	15	5.05 ± 0.01 ^a^	0.11 ± 0.01 ^a^	4.51 ± 0.06 ^b^
T5	80	60	5.06 ± 0.01 ^a,d^	0.10 ± 0.00 ^a^	4.78 ± 0.00 ^b^
T6	80	300	5.07 ± 0.01 ^a,d^	0.11 ± 0.00 ^a^	4.91 ± 0.06 ^a^
T7	90	15	5.12 ± 0.02 ^b,e^	0.11 ± 0.01 ^a^	4.80 ± 0.00 ^a,b^
T8	90	60	5.14 ± 0.02 ^b^	0.11 ± 0.01 ^a^	5.07 ± 0.06 ^a^
T9	90	300	5.09 ± 0.01 ^d,e^	0.11 ± 0.00 ^a^	5.07 ± 0.06 ^a^

Results are expressed as the mean ± standard deviation. Different letters within the same parameter indicate significant differences (*p* < 0.05). TA: total acidity (g of citric acid per 100 mL of sample); TSS: total soluble solids; N/A: not applied.

**Table 2:** Total phenolic content, total anthocyanins, and antioxidant capacity of açaí pulp–whey-based beverages (APWBB) subjected to different thermal treatments.

Treatment	Temp	Time	TPC	TAC	FRAP	DPPH	ABTS
	°C	s	mg GAE/100 mL	mg/100 mL	µmol Fe_2_SO_4_/mL	µmol TEAC/mL	µmol TEAC/mL
Açaí pulp	N/A	N/A	75.51 ± 2.14	14.95 ± 0.14	41.38 ± 0.24	7.21 ± 0.02	8.49 ± 0.08
Control	Unheated	N/A	44.30 ± 2.92 ^a^	10.03 ± 0.15 ^a^	27.15 ± 0.19 ^a^	3.92 ± 0.12 ^a^	4.68 ± 0.08 ^a^
Treatments							
T1	75	15	41.60 ± 3.65 ^a^	9.46 ± 0.10 ^b^	25.01 ± 0.31 ^b^	3.22 ± 0.04 ^b^	4.43 ± 0.08 ^b^
T2	75	60	41.27 ± 3.55 ^a^	9.20 ± 0.11 ^b^	24.30 ± 0.24 ^b,c^	2.95 ± 0.06 ^c^	4.17 ± 0.08 ^b,c^
T3	75	300	39.24 ± 3.25 ^a^	8.80 ± 0.11 ^c^	23.65 ± 0.46 ^c^	2.88 ± 0.07 ^c,d^	4.06 ± 0.08 ^c^
T4	80	15	28.11 ± 0.59 ^b^	7.44 ± 0.06 ^d^	20.89 ± 0.33 ^d^	2.82 ± 0.05 ^d,e^	3.73 ± 0.04 ^d^
T5	80	60	24.73 ± 0.58 ^bc^	7.30 ± 0.09 ^d^	20.34 ± 0.51 ^d^	2.76 ± 0.02 ^e,f^	3.63 ± 0.04 ^d^
T6	80	300	23.38 ± 0.00 ^bc^	7.24 ± 0.05 ^d^	20.13 ± 0.15 ^d^	2.63 ± 0.06 ^f^	3.43 ± 0.04 ^e^
T7	90	15	21.36 ± 1.75 ^cd^	7.11 ± 0.03 ^d,e^	18.49 ± 0.59 ^e^	2.60 ± 0.02 ^f^	3.35 ± 0.04 ^e,f^
T8	90	60	18.66 ± 2.34 ^cd^	6.90 ± 0.10 ^e^	18.12 ± 0.11 ^e^	2.51 ± 0.02 ^f,g^	3.23 ± 0.04 ^f,g^
T9	90	300	16.30 ± 0.00 ^d^	6.30 ± 0.07 ^f^	18.08 ± 0.38 ^e^	2.42 ± 0.02 ^g^	3.08 ± 0.07 ^g^

Results are expressed as the mean ± standard deviation. Different letters indicate significant differences (*p* < 0.05) according to Tukey’s HSD multiple range test. Temp: temperature; s: seconds; TPC: total phenolic content; GAE: gallic acid equivalent; TAC: total anthocyanin content; TEAC: Trolox equivalent antioxidant capacity; N/A: not applied.

## Data Availability

The original contributions presented in the study are included in the article. Further inquiries can be directed to the corresponding authors.
